# Appraisals of Bangladeshi Medicinal Plants Used by Folk Medicine Practitioners in the Prevention and Management of Malignant Neoplastic Diseases

**DOI:** 10.1155/2016/7832120

**Published:** 2016-01-14

**Authors:** Md. Nur Kabidul Azam, Md. Mizanur Rahman, Samanta Biswas, Md. Nasir Ahmed

**Affiliations:** ^1^Ethnobotany & Ethnomedicine Division, TechB Herbal Solution, Kushtia 7040, Bangladesh; ^2^Medical College for Women & Hospital (MCW&H), Uttara Model Town, Dhaka 1230, Bangladesh

## Abstract

Cancer is a group of diseases which is categorized to differentiate into diverse cell types and move around in the body to sites of organogenesis that is key to the process of tumor genesis. All types of cancer fall into the group of malignant neoplastic diseases. In Bangladesh, cancer is now one of the foremost killer diseases and its personal, social, and economic bearing are huge. Plant-derived natural compounds (vincristine, vinblastine, etoposide, paclitaxel, camptothecin, topotecan, and irinotecan) are useful for the treatment of cancer. Since there is no extensive ethnobotanical research study in Bangladesh regarding the traditional uses of medicinal plants against neoplasms, therefore, a randomized ethnopharmacological surveys were carried out in 3 districts of Bangladesh to learn more about the usage of anticancer medicinal plants and their chemical constituents having antineoplastic activity. Comprehensive interviews were conducted to the folk medicine practitioners and medicinal plants as pointed out by them were photographed, collected, deposited, and identified at the Bangladesh National Herbarium. The various plant parts have been used by the healers which included whole plant, leaves, fruits, barks, roots, and seeds. This study evaluated considerable potential for discovery of novel compounds with less side effects in the management and prevention of malignancy in cancer.

## 1. Introduction

Cancer is defined as an abnormal growth of cells caused by multiple changes in gene expression leading to deregulated balance of cell proliferation and cell death. Cancer is those tumors [1] that have developed the ability to invade the surrounding normal tissues. Cancers are caused by exogenous chemical, physical, or biological carcinogens in humans and the mechanisms of carcinogenesis are often multifactorial and complex. Different factors may act by different mechanisms and at different stages of tumor development [2]. A cancerous cell is traveling throughout the body using the blood or lymph systems, destroying healthy tissue in a process called invasion, and that cell manages to make new blood vessels to feed itself in a process called angiogenesis. Tumors may activate angiogenic inhibitors (angiostatin and endostatin) that can modulate angiogenesis at both the primary site and downstream sites of metastasis [3, 4], when a tumor successfully spreads to other parts of the body using the blood or lymph systems known as metastasis.

Cancer is a leading cause of death in the western world. In the United States and a number of European countries, cancer is the second leading destroyer after cardiovascular diseases [5]. Cancer can occur at any age and the average age at the time of diagnosis for cancer is 67 years, and about 76% of all cancers are diagnosed at the age of 55 or older. Although cancer is relatively rare in children, it is the second leading cause of death in children ages of 1–14. In this age, leukemia is the most common cause of death. The overall death rates due to cancer have almost tripled since 1930 for men and gone up over 50% for women [6]. World Health Organization (WHO) estimates that some 84 million people will die of cancer between 2005 and 2015 around the world. In 2007, there were 7.9 million deaths from cancer, around 13 percent of all deaths.

### 1.1. Cancer Epidemiology in Bangladesh

The National Institute of Cancer Research and Hospital (NICRH) started a cancer registry in 2005 for the first time in Bangladesh along with the World Health Organization (WHO). This report covers three years from 2005 to 2007. Data were collected from 24,847 cancer patients who appeared in the NICRH for the first time [7]. Among them, 10,847 (57.6%) were males. Lung cancer was the leading cancer (17.3%), followed by cancers of breast (12.3%), lymph nodes and lymphatics (8.4%), and cervix (8.4%) for sexes combined in all ages. In males' lung (25.5%) and in females breast (25.6%) and cervical (21.5%) cancers were predominant. In children aged 14 years or younger, lymphoma, retinoblastoma, osteosarcoma, leukemia, and kidney cancers were most prevalent. Lung cancer in males and cervical and breast cancer in females constitute 38% of all cancers in Bangladesh [7]. According to the WHO data published in April 2011, oral cancer deaths in Bangladesh reached 11,562 or 1.21% of total deaths. The age adjusted death rate is 12.52 per 100,000 of population ranking Bangladesh 4 in the world. There are more than one million (10 lakh) cancer patients in Bangladesh while approximately 200,000 new patients, mostly women, are added every year creating a social burden on the country [8, 9].

Various plants have been used against cancer and tumor in traditional medicine system of Bangladesh since many years. Traditional medicinal knowledge has been a means towards the discovery of many modern medicines [10]. Traditional medicine is practiced in Bangladesh by folk medicine practitioners, also known as* Kabirajes* who utilize various formulations of medicinal plants in most of their preparations. We have observed that the* Kabirajes* of various districts and areas use diverse varieties of plants for the treatment of schizophrenia and psychotic problems [11], cardiovascular problems [12], eye infections [13], snakebite [14], diabetes [15], gastrointestinal disorders [16, 17], HIV/AIDS related infections [18], rheumatoid arthritis [19], cattle diseases [20], and so on.

It was objective of the present study to conduct a randomized ethnopharmacological survey to learn more about the medicinal plants used by folk medicine practitioners of Bangladesh for the treatment of cancer and also to do comprehensive study on several published articles attributed to the* in vivo* or* in vitro* anticancer properties of these species. The anticipation was that the medicinal plants used by the* Kabirajes* can prove to be a useful source for further scientific studies leading to discovering more efficacious antineoplastic drugs.

## 2. Methodology

### 2.1. Geographical Location of the Survey Area

**Figure 1:**
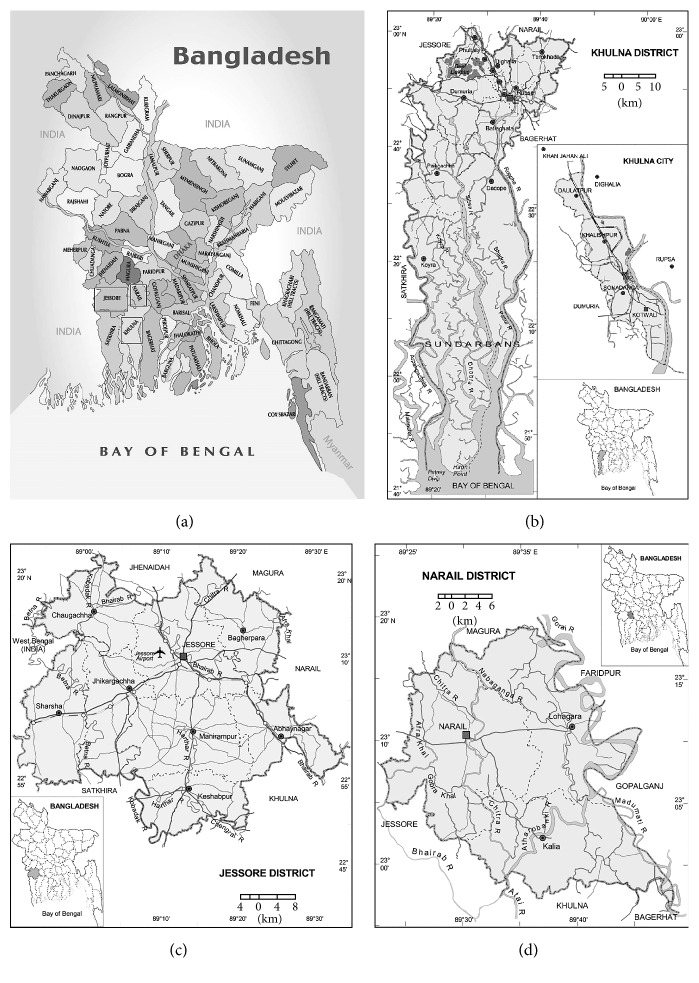
(a) Map of Bangladesh showing survey area with square shade: (b) Khulna district, (c) Jessore District, and (d) Narail District.

The present randomized surveys were carried out between October 2013 and March 2014, among the* Kabirajes* of three districts of Bangladesh, namely, Jessore, Khulna, and Narail. Jessore district geographically is in the southwestern region of Bangladesh. It is located at 23°10′0′′ North, 89°13′0′′ East, bordered by Khulna and Satkhira district to the south, India to the west, Magura and Narail district to the east, and Jhenaidah district to the north. Khulna and Narail district geographically coordinate at 22°48′0′′ North, 89°33′0′′ and 23°10′0′′ North, 89°30′0′′ East, respectively. These three districts (Figure 1) are a part of Khulna division.

The surveys were conducted with the help of a semistructured questionnaire and the guided field-walk method [21, 22]. A total of 5* Kabirajes* (36–60 years) were interviewed during the surveys.* Kabirajes* were asked whether they know about cancer and whether they treat the cancer on a regular basis.* Kabirajes* were selected based on their confirmatory answer to both questions. The* Kabirajes* mentioned the plants with which they treated cancer and took the interviewers to spots from where they collected the plants. All interviews were conducted in the Bangla language. The plants were shown along with providing of local names and the parts used. Plant specimens were collected and dried in the field and later brought back to Dhaka for complete identification at the Bangladesh National Herbarium. Nomenclature of the identified species was documented from the plant list database [http://www.theplantlist.org/].

## 3. Results

**Figure 2:**
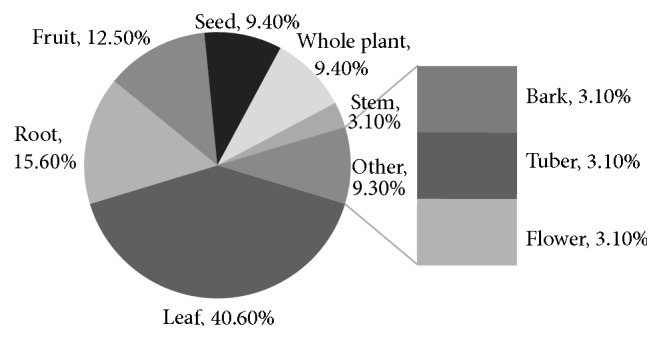
Percentage of plant parts used by the traditional medicine practitioners in the prevention and management of cancer.

A total of 20 plant species were obtained from the* Kabirajes* of the three districts surveyed. The results are summarized in Table 1. These plant species are wild and belonged to 17 families. The Acanthaceae, Cucurbitaceae, and Fabaceae family contributed two plants each; the rest of the families contributed one plant each. Whole plant as well as plant parts like leaves, barks, roots, fruits, and seeds was used for preparing medicine. Leaves constituted the major plant part used, forming 40.6% of total uses. Roots, fruits, and seeds each constituted accordingly 15.6%, 12.5%, and 9.4% of total uses. The other plant parts (whole plant, stem, bark, flower, and tuber) mentioned constituted, respectively, 9.4 and 3.1% of total uses (Figure 2).

### 3.1. Types of Cancer

Among developed countries, the incidence and mortality rates for various cancers are almost the same. Lung cancer is the most common cancer among men in both developing and developed countries of the world and breast cancer is the most common cancer in women. Annually, the global death rate for cancer is estimated to be more than 6 million people and over 22 million individuals have been diagnosed with cancer worldwide [23].  Table 2 has listed the types of cancer.

## 4. Discussion

Many developing countries have intensified their efforts in documenting the ethnomedical data and scientific literature on medicinal plants. In 2000, natural product derivatives were involved in 14 of the top 35 drugs based on worldwide sales [24]. Cancer chemoprevention with phytochemical compounds is a developing plot [25]. Medicinal plants have been used for cancer treatment in many countries of the world from a prolonged period of time [26, 27] and the treatment or prevention is attributed to their safety, low cost, and oral bioavailability as well; natural plant derivatives claimed extensive scientific screening and clinical experiments for the development of anticancer drugs [28]. Over 3000 plants species have been reported to have anticancer properties [29] and about 35000 plant samples from 20 countries have been collected and around 114,000 extracts were screened against tumor systems used as a primary screen [30]. Clinically active antineoplastic agents should be able to prolong the survival and decrease the leukocyte count of blood of tumor-bearing animals [31]. Examples of some well-known plant-derived antineoplastic lead compounds along with their specific mechanism of actions are summarized in Table 3.

### 4.1. Appraisement of Bangladeshi Medicinal Plants Used by the Folk Medicine Practitioners for Antineoplastic Properties

Secondary metabolites are compounds belonging to varied chemical groups that exert biological activities both on human and animal cells. Products of secondary metabolites are the main phytochemical constituents with various pharmaceutical properties serving either as protective agents against various pathogens or growth regulatory molecules. These physiological functions are the effects on cancer cells or tumor development inhibition. Plant-derived commercial anticancer drugs (vinblastine and vincristine from* Catharanthus roseus*) are still produced by isolation from growing plants [32]. In Table 4, we have listed some reported plant-derived chemical compounds from the antineoplastic plants used by the Bangladeshi folk health practitioners in the treatment of cancer.

### 4.2. *Abelmoschus moschatus* (Musk Mallow)

Hydroalcoholic seed and leaf extracts of* Abelmoschus moschatus* exhibited antiproliferative activity against colorectal adenocarcinoma and retinoblastoma human cancer cell lines [33].

### 4.3. *Acanthus ilicifolius* (Holly Mangrove)

The ethanol leaves' extract of the plant was found [34] to be cytotoxic towards lung fibroblast cells in MTT assay. Another study [35] reported that the plant extract has been shown to prevent DNA alterations in a transplantable Ehrlich Ascites carcinoma-bearing murine model and in enlargement of the survival of the animals against the proliferation of ascites tumor. Ethyl acetate extract of the whole plant of* A. ilicifolius* has a potential cytotoxic activity on HeLa cell and KB cell lines by comet assay [36]. Active compounds of* A. ilicifolius* flower play a role in killing* Artemia salina* nauplii and can be considered as potential cytotoxic agents as well as future candidate for cancer therapy [37].

### 4.4. *Aristolochia indica* (Indian Birthwort)

The cytotoxicity and antitumor activity of the chloroform extracts of* Aristolochia indica* were assessed in human breast cancer cell line by MTT assay using taxol as standard and showed pronounced anticancer activity against Ehrlich Ascites Carcinoma cell line [38, 39]. Aristoloside compound was reported to inhibit carcinogenesis [40]. Aristolochic acid was reported to possess various biological activities including antiadenocarcinoma, antineoplastic [41], and antitumor activities [42].

### 4.5. *Borassus flabellifer* (Asian Palmyra Palm)

Dammarane triterpenoid 1, isolated from* Borassus flabellifer* seed coat, inhibits tumor necrosis factor-*α* and showed good antiproliferative activity against pancreatic cancer cell line. Apoptosis inducing activity was confirmed based on increased sub-G0 phase cell population in cell cycle analysis, loss of mitochondrial membrane potential, elevated levels of cytochrome c, nuclear morphological changes, and DNA fragmentation in MIA PaCa-2 pancreatic cancer cells [43].* B. flabellifer* seed coat extracts were screened in another study [44] for their possible anticancer activity on growth of the HeLa cells and these preliminary studies indicated that even the lower concentrations of plant extract showed significant antiproliferative activity.

### 4.6. *Blumea lacera* (Blumea)

There is an* in vitro* study [45] that showed that* Blumea lacera* exhibited broad spectrum antileukemic activity against K562, L1210, P3HR1, and U937 leukemia cells. Methanolic extract of* B. lacera* leaves has also showed cytotoxic activity against human gastric adenocarcinoma cell line, human colorectal adenocarcinoma cell line, and human breast ductal carcinoma cell line [46].

### 4.7. *Cannabis sativa* (Hemp)

The interest in anticarcinogenic properties of cannabinoids was renewed after the discovery of the endocannabinoid system [47]. The administration of Δ9-THC, Δ8-THC, and cannabinol inhibited the growth of Lewis lung adenocarcinoma cells* in vitro* as well as* in vivo* after oral administration in mice [48]. Antitumorigenic mechanisms of cannabinoids are showing their ability to interfere with tumor neovascularization, cancer cell migration, adhesion, invasion, and metastasis [49]. The mechanism of cannabinoids' anticancer action depends on the ability of their agents to stimulate autophagy-mediated apoptotic cancer cell death; thus, cannabinoid action helps in cancer cell death, impairs tumor angiogenesis, and blocks invasion and metastasis [50] and cannabinoids are currently also being tested as anticancer agents in phase I/II clinical studies [51].

### 4.8. *Cucurbita maxima* (Pumpkin)

Methanol extract of* Cucurbita maxima* aerial parts has been performed against Ehrlich Ascites Carcinoma model in mice by Saha et al. [52] for the antitumor activity and the results revealed that* C. maxima* possesses significant anticancer activity which may be due to its cytotoxicity and antioxidant properties. L-asparaginase is an antineoplastic agent, identified from fruit of* C. maxima*, used for treatment of a type of cancer that is acute lymphoblastic leukemia and non-Hodgkin's lymphoma [53] as well as being experimentally used as an anticancer agent in human patients [54, 55].

### 4.9. *Dillenia indica* (Elephant Apple)

Leaf powder of* Dillenia indica* is given in treatment of breast cancer [56]. The methanolic extract of* D. indica* has been found to have significant antileukemic activity in human leukemic cell lines U937, HL60, and K562 [57]. Methanolic extracts of betulinic acid were prepared from the* D. indica* fruits inducing apoptosis in HT-29 cells via mitochondrial dependent pathway and proving to be a potential therapeutic agent for colon cancer [58].

### 4.10. *Dioscorea bulbifera* (Air Potato)

Petroleum ether fraction of the plant showed potential effects against HepA with microstructure abnormality of HepA cells surface [59]. Immune system modulation might be related to antitumor effects of* D. bulbifera* rhizome, as reported in S180 and H22 tumor cells bearing mice [60].

### 4.11. *Emilia sonchifolia *(Lilac Tasselflower)

The aqueous and methanolic extracts of the leaves of* Emilia sonchifolia* gradually exhibit antitumor activities [61]. The n-hexane extract of* E. sonchifolia* has anticancer effect and is rich in terpenoids [62] and terpenoids were evaluated for their potential antineoplastic activity in various human cancer cell lines such as gastric, pancreatic, and colon carcinomas [63].

### 4.12. *Erythrina variegata* (Tiger's Claw)

Steroid derived from the stem bark and the leaves of* Erythrina variegata* showed anticancer activity against* in vitro* breast cancer cell T47D [64]. Alkaloids (10,11-dioxoerythratidine and crystagallin A) extracted from the leaves and stem bark of* E. variegata* plant strongly stated* in vitro* anticancer activity against breast cancer T47D cell lines* in vitro* using the Sulforhodamine B (SRB) assay [65].

### 4.13. *Hygrophila auriculata* (Marsh Barbel)

The effect of* H. auriculata* on carbohydrate metabolizing enzymes in N-nitrosodiethylamine induced hepatocellular carcinoma in rats [66]. The aqueous seed extract from* H. auriculata* displayed selective cancer cell cytotoxicity with an IC50 value of 0.22 mg mL^−1^ against colon cancer cells [67].* In vitro* study of* H. auriculata* extracts has reported antitumor and NF*κ*B inhibition [68]. Ahmad et al. reported antitumor activity from plant extract against chemically induced hepatocarcinogenesis in Wister rats [69].

### 4.14. *Moringa oleifera* (Drumstick Tree)

A hydroalcoholic extract of* Moringa oleifera* study revealed possible chemopreventive potential against chemical carcinogenesis [70]. Different leaf extracts of* M. oleifera* produced significant cytotoxic effects on human multiple myeloma cultured cell lines [71]. A study [72] showed that leaves extract of* M. oleifera* can significantly obstruct the growth of cultured human pancreatic carcinoma cells by inhibiting the NF-*κ*B signaling pathway. Most of the anticancer studies of* M. oleifera* have not focused on the molecular basis of the tumor-suppressive activity but strongly suggested that it could potentially be a supreme anticancer candidate specific to cancer cells [73, 74].

### 4.15. *Nymphaea nouchali* (Blue Water Lily)

The methanolic extract of* Nymphaea nouchali* roots has showed inhibitory activity towards tumor promoter in the Raji cells [75].

### 4.16. *Persicaria hydropiper* (Water Pepper)

*In vitro* antiproliferative activity of* Polygonum hydropiper* (synonymy) extracts was evaluated against cervix epithelial adenocarcinoma, skin epidermoid carcinoma, and breast epithelial adenocarcinoma cells and the results confirmed substantial cell growth inhibitory activity against one or more cell lines [76].

### 4.17. *Trichosanthes kirilowii* (Chinese Cucumber)

A triterpenoid compound named cucurbitacin B isolated from* Trichosanthes kirilowii* showed the potent inhibitory activity against HIF-1 activation induced by hypoxia in various human cancer cell lines.* In vivo* studies confirmed the inhibitory effect of cucurbitacin B on the expression of HIF-1*α* proteins, leading to a decrease growth of HeLa cells in a xenograft tumor model [77]. Cucurbitacin D isolated from the plant has also been shown to suppress proliferation of HT-29 human colon cancer cells [78] and the compound could be potent therapeutic agent for breast cancer by blocking tumor cell proliferation and inducing apoptosis through suppression of STAT3 activity [79] and it could also induce apoptosis in human hepatocellular carcinoma cells [80].

## 5. Conclusions

Among twenty plant species, four of the species used by folk medicine practitioners have no strong published data regarding anticancer or cytotoxic activities. These 4 species are* C. inerme*,* M. paniculata*,* S. sesban*, and* V. officinalis*. From just a brief survey of the literature, it appears that the rest of the sixteen plants used by the* Kabirajes* in three districts of Bangladesh present considerable potential in the treatment of cancer. Further scientific studies need to be conducted on these plants towards discovery of lead compounds, which can lead to formulation of new drugs for the prevention and management of malignant neoplastic diseases with giving less or no side effects.

## Figures and Tables

**Table 1:** Medicinal plants used by the folk medicine practitioners in three districts of Bangladesh for prevention and management of malignancy in cancer.

Serial number	Botanic name	Family name	Vernacular name	Part(s) utilized
1	*Acanthus ilicifolius* L.	Acanthaceae	Harjora	Leaf
2	*Hygrophila auriculata *(Schumach.) Heine	Acanthaceae	Makhna	Leaf
3	*Borassus flabellifer* L.	Arecaceae	Tal	Root, fruit
4	*Aristolochia indica* L.	Aristolochiaceae	Ichamul	Leaf
5	*Emilia sonchifolia* (L.) DC. ex DC.	Asteraceae	Shadhi	Whole plant
6	*Cannabis sativa* L.	Cannabaceae	Bhang	Leaf, root
7	*Blumea lacera* (Burm. f.) DC.	Compositae	Kukurshunga	Leaf
8	*Trichosanthes kirilowii* (Maxim.) Kuntze	Cucurbitaceae	Lotaakal	Whole plant
9	*Cucurbita maxima* Duchesne	Cucurbitaceae	Kumra	Leaf, stem, and fruit
10	*Dillenia indica* L.	Dilleniaceae	Chalta	Leaf, fruit
11	*Dioscorea bulbifera* L.	Dioscoreaceae	Lota-bori	Root, fruit
12	*Erythrina variegata* L.	Fabaceae	Mandar gach	Leaf
13	*Sesbania sesban* (L.) Merr.	Fabaceae	Dhoinche	Leaf, bark, flower, and seed
14	*Abelmoschus moschatus* Medik.	Malvaceae	Lota koshturi	Leaf, seed
15	*Moringa oleifera* Lam.	Moringaceae	Shajna	Leaf
16	*Nymphaea nouchali* Burm. f.	Nymphaeaceae	Shapla	Tuber, root
17	*Persicaria hydropiper* (L.) Delarbre	Polygonaceae	Bishalo-pata	Leaf, seed
18	*Murraya paniculata* (L.) Jack	Rutaceae	Kamini gach	Leaf
19	*Veronica officinalis* L.	Scrophulariaceae	Chapta-pata	Whole plant
20	*Clerodendrum inerme* (L.) Gaertn.	Verbenaceae	Jongli jui	Root

**Table 2:** List of cancer types [23].

Lung cancer	Breast cancer

Colorectal cancer	Liver cancer

Pancreatic cancer	Cancers of the female reproductive tract (cervical cancer, endometrial cancer, and ovarian cancer)

Prostate cancer	Urinary bladder cancer

Lymphoma	Leukemia

Skin cancer	Cancer of the central nervous system

**Table 3:** List of some plant-derived antineoplastic lead compounds currently in use and currently in clinical trials [81, 82].

	Source of plant	Specific mechanism of actions of the lead compounds
Antineoplastic lead compounds currently in use		
Vinblastine, Vincristine	*Catharanthus roseus*	Bind to the microtubulin site in the *β*-subunit and disrupt the assembly of microtubules in mitosis [83]
Taxol	*Taxus brevifolia*	Binds to the taxane site as a microtubule stabilizer and interfering with the normal breakdown of microtubules during cell division [84]
Etoposide	*Podophyllum peltatum*	Binds to tubulin and interferes with the formation of spindles in mitosis [85]
Camptothecin, irinotecan, and topotecan	*Camptotheca acuminata*	Arrest the cell cycle at the S-phase by inhibiting the activity of topoisomerase I, leading to the inhibition of DNA replication and transcription [86, 87]
Antineoplastic lead compounds currently in clinical trials		
Homoharringtonine	*Harringtonia cephalotaxus*	Inhibits protein synthesis and blocking cell-cycle progression [88], promotes apoptosis, and inhibits protein synthesis at the ribosomal level [89, 90]
Curcumin	*Curcuma longa*	Induces apoptosis and inhibits the proliferation of a variety of malignant cells and is involved in the regulation of combined signaling pathways at multiple levels by acting on various targets including modulation of gene transcription factors (NF*κ*B, p53, and AP-1), growth factors and their receptors (PDGF, EGF, and VEGF), cell surface adhesion molecules (E-cadherin, *β*-cadenin), and protein kinases (CDKs, EGFR, PKC, and p38 MAPK) [91, 92]
Resveratrol	*Vitis vinifera, Morus alba, *and* Arachis hypogaea*	Inhibits the growth of cancer cells and induces apoptosis by acting at multiple cellular targets, including activation of p53, inhibiting 10 otulins, 10 genases, and cytochrome P450 enzymes, and activating AMP-activated kinase (AMPK) [93–95]
Flavopiridol	*Amoora rohituka*	Exhibits apoptosis induction [96], inhibits the activity of cyclin-dependent kinases (CDKs) by competing with ATP at their nucleotide binding sites, and causes cell cycle arrest at either the G1 or G1/M phases [97]

**Table 4:** List of reported phytochemicals from Bangladeshi antineoplastic plants used by folk medicine practitioners.

Serial number	Plant source	Phytochemical constituents	Reference
1	*Abelmoschus moschatus*	Uridine (1-[(3R,4S,5R)-3,4-dihydroxy-5- (hydroxymethyl)oxolan-2-yl]pyrimidine- 2,4-dione), n-tridecane, isopentyl 2-methyl butanoate, and decanal	[98, 99]

2	*Acanthus ilicifolius*	Flavonoids, glycosides, saponins, steroids, and tannins; lupeol, *α*-amyrin, olcanolic acid and ursolic acids; saponin and triterpenoid saponin; steroids (stigmasterol, campesterol, and sitosterol); alkaloids (acanthicifoline and benzoxazinium); and phenolics (acanfolioside, ilicifolioside, acteoside, verbascoside, and apigenin)	[100–104]

3	*Aristolochia indica*	Aristolochic acid, flavonoids, tannins, glycosides, phenol, and saponins	[38, 105]

4	*Blumea lacera*	Thymoquinol dimethyl, *β*-caryophyllene, *α*-humulene, and E-*β*-farnesene; 5-hydroxy-3,6,7,3′,4′-pentamethoxy flavone, 5,3′,4′-trihydroxy flavone	[46, 106]

5	*Borassus flabellifer*	Dammarane triterpenoid; resorcinol, phenol, pentanoic acid, glycerin, 10-undecenyl ester, octadecanoic acid, and n-hexadecanoic acid	[43, 107]

6	*Cannabis sativa*	Cannabinoids; Δ9-THC, Δ8-THC	[48, 108]

7	*Clerodendrum inerme*	Neoclerodane diterpenoids (inermes A, B and 14,15-dihydro-15*β*-methoxy-3-epicaryoptin), megastigmane glycosides, and iridoid glycoside	[109, 110]

8	*Cucurbita maxima*	Carbohydrates, alkaloids, glycosides, tannins, flavonoids, and saponins steroids; L-asparaginase; glutamic acid, calcium, and resin; and *β*-carotene, lycopene, and lutein	[53, 111–113]

9	*Dillenia indica*	Dihydroisorhamnetin, dillenetin; tannin, betunaldehyde, betulinic acid, rhamnetin, dihydroisorhamnetin, lupeol, myricetin, naringenin, quercetin and kaempferol glucoside, and stigmasterol	[114, 115]

10	*Dioscorea bulbifera*	Kaempferol-3, 5-dimethyl ether, caryatin, (L)-catechin, myricetin, quercetin-3-O-galactopyranoside, myricetin-3-O-galactopyranoside, myricetin-3-O-glucopyranoside, and diosbulbin B	[116]

11	*Emilia sonchifolia*	Beta-sitosterol, stigmasterol, palmitic acid, and honey acid	[117]

12	*Erythrina variegata*	Lectin, isoflavones, alkaloids, flavonoids, pterocarpans, triterpenes, steroids, alkyl transferulates, proteins, lecithin, 10,11-dioxoerythratidine, and crystagallin A	[65, 118–120]

13	*Hygrophila auriculata*	Flavonoids and polyphenols; apigenin 7-O-glucuronide alkaloids (asteracanthine and asteracanthicine); triterpenes (lupeol, hydrocarbon, hentriacontane, 13 otulin, luteolin, and luteolin-7-O-rutinoside); aliphatic esters (25-oxo-hentriacontyl acetate, methyl 8-nhexyltetracosanoate); and sterols (stigmasterol)	[121–126]

14	*Moringa oleifera*	Flavonoid pigments (kaempferol, rhamnetin, isoquercitrin, and kaempferitrin), glycoside compounds, glucosinolates, and isothiocyanates; beta-sitosterol, glycerol-1-(9-octadecanoate), 3-O-(6′-O-oleoyl-beta-D-glucopyranosyl), beta-sitosterol, and beta-sitosterol-3-O-beta-D-glucopyranoside	[127, 128]

15	*Murraya paniculata*	Coumarins (7-methoxy-8-(3-methyl-2- oxobutoxy)-2H-chromen-2-one, umbelliferone, and scopolin); indole alkaloids (murrayacarine and murrayaculatine)	[129–132]

16	*Nymphaea nouchali*	Protein, carbohydrate, reducing sugar, glycosides, phenol, tannin, flavones, saponin, steroid, alkaloid, anthraquinone, quinone, and lectin	[133, 134]

17	*Persicaria hydropiper*	Apigenin-7-O-glucoside, catechin, epicatechin, hyperin, isoquercitrin, kaempferol, kaempferol rutinoside, quercitrin, persicarin, rhamnetin, polygonic acid, polygodial acetal	[135]

18	*Sesbania sesban*	Oleanolic acid, stigmastane-5.24(28)-diene-3*β*-O-*β*-D-galactopyranoside, galactomannan, phenols, flavonoids and anthocyanins, and saponin	[136–138]

19	*Trichosanthes kirilowii*	Cucurbitacin B, cucurbitacin D, 4′,6-dihydroxy-4-methoxyisoaurone	[77, 139, 140]

20	*Veronica officinalis*	Terpenes, esters, steroids (sterols and sterenes), p-hydroxyphenyl ethyl alcohol, maltol, and loliolide. *β*-sitosterol; *α*-linolenic and linoleic acid; and iridoid glucoside	[141, 142]
